# Cost-Effectiveness Analysis of Total Hip Arthroplasty Performed by a Canadian Short-Stay Surgical Team in Ecuador

**DOI:** 10.1155/2017/5109895

**Published:** 2017-12-18

**Authors:** Michael Schlegelmilch, Saifee Rashiq, Barbara Moreau, Patricia Jarrín, Bach Tran, Anderson Chuck

**Affiliations:** ^1^Faculty of Medicine and Dentistry, University of Alberta, Edmonton, AB, Canada; ^2^Canadian Association of Medical Teams Abroad, Edmonton, AB, Canada; ^3^Department of Anesthesiology and Pain Medicine, University of Alberta, Edmonton, AB, Canada; ^4^Fundación Tierra Nueva, Quito, Ecuador; ^5^Institute of Health Economics, Edmonton, AB, Canada

## Abstract

**Background:**

Few charitable overseas surgical missions produce cost-effectiveness analyses of their work.

**Methods:**

We compared the pre- and postoperative health status for 157 total hip arthroplasty (THA) patients operated on from 2007 to 2011 attended by an annual Canadian orthopedic mission to Ecuador to determine the quality-adjusted life years (QALYs) gained. The costs of each mission are known. The cost per surgery was divided by the average lifetime QALYs gained to estimate an incremental cost-effectiveness ratio (ICER) in Canadian dollars per QALY.

**Results:**

The average lifetime QALYs (95% CI) gained were 1.46 (1.4–1.5), 2.5 (2.4–2.6), and 2.9 (2.7–3.1) for unilateral, bilateral, and staged (two THAs in different years) operations, respectively. The ICERs were $4,442 for unilateral, $2,939 for bilateral, and $4392 for staged procedures. Seventy percent of the mission budget was spent on the transport and accommodation of volunteers.

**Conclusion:**

THA by a Canadian short-stay surgical team was highly cost-effective, according to criteria from the National Institute for Health and Care Excellence and the World Health Organization. We encourage other international missions to provide similar cost-effectiveness data to enable better comparison between mission types and between mission and nonmission care.

## 1. Background

In the past decade, osteoarthritis (OA) had the largest increase of associated disability adjusted life years (DALYs) globally and was the tenth leading cause of years of life lost due to a disability [1]. Recent world data suggest that deaths from rheumatic diseases are also increasing [2]. Total hip arthroplasty (THA) is the definitive treatment for end-stage degenerative hip disease, but many cultural and socioeconomic barriers to accessing THA, such as race, exist worldwide [3]. Individuals in low- and middle-income countries (LMICs) are disproportionately impeded by these barriers and are thus largely denied the opportunity to receive THAs. Given the dependence that many of them have on manual labor for subsistence, the inaccessibility of treatment can have a large economic impact.

Charitable donations to organizations that deliver medical and surgical care in LMICs are common in wealthier nations. In 2010, Canadian charities received more than $1.5 billion in donations to support medical work of all kinds, of which a subset is earmarked for short-stay surgical missions [4]. While all charities publish the ways in which donor funds are spent, very few provide cost-effectiveness analyses of their interventions.

The Canadian Association of Medical Teams Abroad (CAMTA) (http://www.camta.com), an Edmonton, Alberta, based charity, conducts annual orthopedic surgical missions to Ecuador to perform THAs in adults and miscellaneous complex orthopedic procedures in children. CAMTA's origins began with the direct observation of shortfalls in access to such care made by an informally constituted team of visiting health providers many years ago. It began operating in Ecuador in 2002, treating early onset hip osteoarthritis attributable to the failed detection or treatment of developmental dysplasia of the hip. It should be noted that CAMTA offers these specific interventions because they lie within the expertise of its members, not because they were prioritized by any assessment of local needs. CAMTA works in the hospitals of Fundación Tierra Nueva (FTN), a health and social welfare charity in Quito. It also provides some educational programing for Ecuadorian orthopedic surgeons. In parallel, it offers other capacity building initiatives not described here.

The purposes of this investigation were to calculate the cost effectiveness of the THA operations that CAMTA performs and to compare this with published cost-effectiveness data for other humanitarian surgical and nonsurgical programs.

## 2. Methods

Approval was obtained from the Health Research Ethics Board, Health Panel of the University of Alberta, to use data routinely collected for quality assurance purposes for this investigation.

FTN (http://www.fundaciontierranueva.org.ec) is a faith-based charity in Quito, Ecuador, that offers social assistance and health care to those who cannot afford to procure it privately. Examples of its activities include ambulatory care, obstetric services, child development support, mobile dental care, and support for those with disabilities. In addition, it operates an acute care hospital with a surgical suite. Here, local surgeons perform general surgery, gynecology, urology, and orthopedics (including THA but not pediatrics). In parallel, overseas surgical teams, of which CAMTA is one, perform specialty surgery on a short-stay basis on a rotating schedule. FTN is supported by a combination of charitable donations and remittances from the government for providing medically necessary services. FTN supplies CAMTA with clinic, operating, and recovery rooms, sterilizing facilities, postoperative beds, some nurses, social workers, administrative and maintenance personnel, laundry, utilities, and piped oxygen. It invoices CAMTA for these items. It charges consumable items at cost and provides the services of its personnel at their usual hourly rate. CAMTA provides all other personnel (surgeons, anesthetists, family physicians, most nurses, physiotherapists, translators, surgical equipment cleaners, and lay helpers) and all other consumables (drugs, dressings, surgical prostheses, and physiotherapy appliances). Personnel numbers are adjusted from year to year depending on workload. CAMTA receives and stores donations of some consumable items in Canada. These are transported in the personal luggage of its participants for each mission at no cost. A small number of items that are deemed mission critical and which are unavailable in Ecuador (principally a few specific anesthesia drugs) are purchased in Canada and transported in the same manner. The remaining necessary consumables are purchased locally. The proportion of supplies acquired locally varies from year to year, depending on the available quantity of donations.

### 2.1. Participant Selection

Patients who are referred from FTN primary care and who might, on clinical grounds, benefit from THA are identified during the year preceding each mission by a specific affiliated Ecuadorian orthopedic surgeon. This group is then means-tested by the Social Work Department of FTN according to its own criteria in order to confirm that they could not otherwise afford the cost of surgery and is then referred to CAMTA. FTN considers household size, total household income, and the number of children or others depending on the earning potential of the patient in order to determine eligibility for CAMTA services, seeking always to prioritize those in the greatest need. It demands at least some contribution from the recipient to the cost of the care it delivers, but this too is means-tested and might be as low as a dollar. (The cost of THA if obtained directly from FTN is $7750, comprising hospital costs of $3400 and prosthesis cost of $4350; about half of Ecuador's population is eligible to have that covered by social insurance, and a further 9% have private insurance, but such an outlay would be beyond the means of the remainder.)

Upon CAMTA's arrival, prospective candidates are screened for surgical appropriateness by Canadian surgeons. Patients are selected for surgery using the same clinical criteria as would have been applied in Canada. Anesthesiologists and family physicians then determine medical fitness for surgery, declining anyone in whom there is a high predictable risk of prolonged recovery or other complications. The costs of any necessary preoperative screening tests are nominally charged to the patient, but the costs of these are included in, not imposed in addition to, FTN's fee. In the event that the number of appropriate candidates exceeds the available surgical capacity in any mission year, surgical patients are selected based on the greatest financial need and most severe health burden. Those who are declined for this reason are invited to return the following year but are not prioritized. Here, we present an analysis of CAMTA's adult patients who underwent surgery from 2007 to 2011.

All patients underwent hip replacement via the posterolateral or lateral approach under spinal anesthesia and conscious sedation, with antibiotic prophylaxis and tranexamic acid administration. This is in keeping with prevailing practices in Canada. Allogeneic blood transfusion was administered as needed. Prophylaxis against deep vein thrombosis for the postdischarge period was provided. The vast majority of patients received uncemented titanium implants unless poor bone quality necessitated cemented implants. Patients were mobilized soon after surgery and discharged on crutches when they could climb a short flight of stairs, typically on postoperative day one. All patients were asked to return to see the affiliated local orthopedic surgeon at six weeks and three and six months. They were asked to return for follow-up with CAMTA one year later.

### 2.2. Health Outcomes

Duly validated Latin American Spanish language versions of two outcome instruments were administered. The SF-36 (Medical Outcomes Study 36-Item Short Form Health Survey) is a widely used instrument for quantifying self-reported quality of life in eight major domains relating to health status [5]. All domains are scored on a scale from 0 to 100, which represent the worst to best possible health states. These results are summarized and presented as a physical component summary (PCS) and a mental component summary (MCS). It was administered preoperatively and at one year. Staged intervention patients were measured for outcomes after each surgery but only reported in this study after their final surgery. The mean of each of the eight domains was calculated each time point. These averages were used for cross-walking onto another instrument called the 15D for the purposes of determining a health utility score.

There were two separate instruments applied to assess patient outcomes during the study period: SF-36 and 15D. The SF-36 was applied pre-op and post-op for patients from 2007 and 2010 and pre-op in 2011. CAMTA began administering the 15D to patients at follow-up in 2012, for patients that received surgery in 2011 and so, in order to group patient data from all five mission years (2007–2011), we cross-walked the SF-36 from each patient onto the 15D. This method has been validated in patients with rheumatoid arthritis [6]. The 15D is a multiattribute utility instrument. Containing a profile of 15 dimensions, it provides a descriptive system of 31 billion discrete states (Table 1) [7, 8]. A set of utility weights of Finland and Denmark is used to generate the 15D score on a 0-1 scale, representing death to perfect health [9]. We rescaled the 15D single score onto a 0–100 scale to ease comparison between the 15D and SF-36. To cross-walk the SF-36, regression analysis was used to examine the relationship between the 15D utility score and 8 domain scores of the SF-36. Three statistical models were used and we found that an ordinary least squares (OLS) model outperformed Tobit and censored least absolute deviations models, as indicated by *R*-squared measure (0.79). Regression coefficients estimated in this OLS model were then selected for deriving utility scores in this study. Quality-adjusted life years (QALYs) gained were calculated based on the percent change in 15D scores for each surgery type over 1 year.

### 2.3. Costs

Costs are from the perspective of CAMTA and all of CAMTA's expenditures are precisely recorded to comply with its registered charity status. CAMTA's Canadian personnel donate their time and cover their own transportation and accommodation by securing donations, which are remitted to CAMTA. Other costs are defrayed by donations to the organization as a whole. During the study period, manufacturers donated most but not all surgical prostheses. We did not include, (but acknowledged) the opportunity costs of personnel who are not working elsewhere and the opportunity costs of not using the surgical and ward space for other uses. Half of CAMTA's patients are children, who were not subjects in this study. Costs attributable to the care of children were excluded from this analysis. All costs are in 2007 Canadian dollars.

### 2.4. Cost Effectiveness

The average cost of each surgery type was determined for unilateral, bilateral, and staged (two THAs in different years) THAs during the study period. Lifetime incremental QALYs gained were estimated with life expectancy tables and were discounted at a rate of 3.5% per year, in accordance with the NICE guidelines [10]. Lifetime QALYs gained and average cost per surgery were used to calculate a point estimate of the incremental cost-effectiveness ratio (ICER). Analyses were performed to determine the sensitivity of the ICER to the two largest categories of expenditures: personnel travel and accommodation and procurement of surgical prostheses.

### 2.5. Ethics and Data Storage and Management

Approval was obtained from the Health Ethics Review Board of the University of Alberta to use data which had been routinely collected as part of the mission years in question for this investigation. The data were stored in sealed boxes to which only senior CAMTA staff had access. Once transcribed onto electronic spreadsheets, they were encrypted and protected by password. Only anonymized data were used in the analysis.

## 3. Results

### 3.1. Enrolment and Demographics

During the five-year study, CAMTA operated on 157 adult patients receiving THA. The majority of patients were female (79%) with an average age of 49 years at the time of their first surgery and an average life expectancy of an additional 32 years, illustrating the comparative precocity of degenerative hip disease in Ecuador compared to Canada. Unilateral THAs comprised 79.6% (*n* = 125) of the total surgeries with 16.6% (*n* = 26) and 3.8% (*n* = 6) of patients receiving bilateral and staged THAs, respectively. Sixty-five patients (41.4%) were lost to follow-up at one year. Of the 92 patients with follow-up data, nearly a third had incomplete responses to the SF-36. There were no significant differences between patients who were included or lost to follow-up based on age or presurgery PCS or MCS score (Table 2).

### 3.2. Health Outcomes

Mean PCS scores improved from 34.2 to 45.6 (<0.001). No significant improvement in MCS scores was seen (*p* = 0.092). Health utility, measured by 15D scores, gained at one year (after the second surgery for staged patients) was the greatest for patients who received the staged intervention (14.1%; 0.141 QALYs), compared to bilateral (12.2%; 0.122 QALYs) and unilateral (10.3%; 0.103 QALYs) intervention.

### 3.3. Costs

The total mission costs for our study period were $1,107,996 CAD. The largest proportion of funds was allocated to transport and accommodation of personnel ($773,947; 70%). Even though the vast majority of the prosthetic joint component pieces were donated without charge, the purchase of the remainder made up the second largest cost at $231,687 (21%). The remaining costs were split between other medical supplies and drugs ($46,307; 4%) and fixed administrative costs (office supplies and equipment, legal and banking fees, and website maintenance) ($56,056; 5%). Mean cost per surgery was lowest for unilateral THA at $6,042, followed by bilateral THA at $7,229. Staged THAs required additional travel and OR costs and had a mean cost of $12,786 (Table 3).

### 3.4. Cost-Effectiveness and Sensitivity Analysis

The ICER for unilateral THAs was $4,443 and that for bilateral and staged THAs was $2,939 and $4,392, respectively (Table 3). Our sensitivity analysis indicates that removing the cost of prostheses would have decreased the ICER for unilateral THA by $1,031 (23%), by $955.17 (32%) for bilateral THA, and by $2,692 (61%) for staged procedures. If CAMTA had paid the full cost of prostheses, the ICER would have risen by $1,072.67 (24%) for unilateral THA, by $1,496.86 (51%) for bilateral surgery, and by $1,104.39 (25%) for staged surgery. Removing the cost of travel and accommodation for CAMTA personnel reduced the ICER 74% to $1,157 for unilateral, by 62% to $1,112 for bilateral, and by 78% to $952 for staged surgeries. In the hypothetical situation where all prostheses were donated free of cost, and excluding personnel costs, ICERs were $402, $234, and $200 for unilateral, bilateral, and staged surgeries, respectively (Table 4). Although we do not have data to propose an ICER for a scenario where Ecuadorian surgeons were performing the THA with prostheses acquired by CAMTA and donated, the final line of Table 4 may approximate this scenario. When no personnel costs were included but full prostheses costs were considered the ICER decreased by $2687.29 (60%) for unilateral, by $1131.52 (38%) for bilateral, and by $2843.42 (65%) for staged surgeries.

## 4. Discussion

We have shown that the mean lifetime cost per QALY for THA in our mission setting is between $2939 and $4442.77 and, depending on the classification of the costs of volunteer transport and accommodation, could be as low as $200.

Our outcomes are well below the threshold of £20,000/QALY ($32,000 CAD/QALY) for cost effectiveness, based on the NICE guidelines [11]. Another, more contextually appropriate, comparator of cost effectiveness uses national GDP. As recommended by World Health Organization, an ICER that is less than the GDP per capita is highly cost-effective [12]. The average GDP per capita in Ecuador during our study period was $4150 CAD [13]. This suggests that, by these definitions, bilateral THAs performed by CAMTA were highly cost-effective. Unilateral and staged interventions exceeded the WHO recommended benchmark by 7% and 6%, respectively.

Humanitarian surgical relief of congenital hand deformities in Honduras and cleft lip in India reported ICERs of $437/DALYs (Disability Adjusted Life Years,* not QALY*) and $247/DALYs, respectively [14, 15]. Orthopedic teams in Haiti ($362/DALYs) and Ecuador ($525/DALYs) and general or obstetrical surgical missions ($308/DALYs) in the Dominican Republic all report similar cost-effectiveness ratios [16–18]. A Médicines Sans Frontières evaluation of two surgical trauma hospitals in Nigeria and Haiti achieved ICERs of $172 and $223, respectively [19]. We reported our outcomes in QALYs gained because 15D is validated for that purpose [8]. In a systematic review of global surgical cost-effectiveness analyses, QALYs were more common, though less so in low- or middle-income countries [20]. Though these comparator studies reported DALYs, theoretical comparisons between QALYs and DALYs can be made, and occasionally QALYs gained will be close to or equal DALYs averted [21]. Although these two measures seek to quantify a similar experience, they are not necessarily reciprocal. The variance between QALY and DALY depends on age, disease duration, comorbid conditions, and the difference between quality of life and disability weights [21, 22]. Even while respecting the cautions that need to be exercised when comparing DALYs and QALYs, our findings are comparable.

An analysis of THAs in the UK reported an ICER of £2852 ($5225 CAD) [23]. However, these authors were able to account for the cost of revisions and complication management. This is a limitation of our study, as we could not account for costs of complications or revisions that CAMTA did not manage. This is a documented issue of many short-stay surgical missions and systematically leads to underestimated cost-effectiveness estimates [24]. We speculate that although complications occur, the rate is low and would not have a significant effect on our outcomes. In Western, tertiary level care settings, the complication rates for a THA 0.5% are in 15 years [23]. In one study of cleft palate repair by a short-stay surgical mission, the complication rate was 20 times greater than rates seen in the USA [25], but, to our knowledge, complication rates have not been compared for THAs.

In contrast, it is possible that for other reasons our study produced more conservative estimates of QALYs gained and overestimated ICERs. First, we were unable to measure the increased lifetime earnings and economic productivity that our patients could achieve after receiving a THA. Second, the quality of life benefits of increased survivability were not accounted for in our QALY calculations. Third, we are limited by attrition, which is a documented issue in the literature. International surgical missions report attrition rates ranging from 20% to 63% [24, 26]. Though we found no difference at baseline between those lost to follow-up and those who were not, we speculate that patients were more likely to be lost if they had favourable surgical outcomes, as they would feel no need to present to clinic. This is on the assumption that complication rates and all-cause mortality are lower than our rates of attrition which are nearly 30%. We assume this is not attributable to poor outcomes, given the previous discussion regarding estimates of complication rates.

Others have hypothesized that inefficiencies in process would produce cost-ineffective interventions when relief teams travel to a different country [16]. Our results suggest otherwise. The sensitivity analysis suggests a remarkably low ICER under circumstances where human resources and prostheses are entirely donated. A $200.68 donation could, in one such hypothetical scenario, procure one QALY for a patient with debilitating hip arthritis.

It is prudent to acknowledge that the perspective of analysis is from that of CAMTA and does not account for costs to patients or to the Ecuadorian health system or the opportunity costs of people and space used for this mission to operate. While CAMTA always welcomes the involvement of Ecuadorian trainees and medical staff in its cases, such involvement is self-initiated. CAMTA does not seek to actively build local capacity as part of its surgical care delivery (but has active parallel initiatives of this type which are not discussed here).

Ecuadorian practitioners are more than capable of performing THA and indeed do so in the very same hospital in which CAMTA operates. Why, then, is the mission needed at all? What would happen if it became impossible for CAMTA to return? We emphatically do not claim that the intermittent arrival of a large group of foreigners is the best way to deliver this care to those who need it, in Ecuador or, by extension, anywhere else, no matter how cost-effective it can be shown to be. Clearly, the optimal scenario would be the timely delivery of such care by local practitioners. What CAMTA brings to bear is essentially the deployment of a high concentration of resources and personnel in a short space of time. Vigorous debate can and must be had at all levels of governmental, professional, charitable, and public bodies about the best way to serve those who need health care of all kinds, in a manner that is accessible, sustainable, and of high quality. All we can hope to do here is to fuel that debate by attaching an approximate dollar value to the clinical results we have achieved.

Though the intrinsic clinical and logistical challenges of medical missions may push the task of outcome data collection out of mind, we concur with Bermudez (2010) in his plea for surgical missions to be evaluated using outcomes and not throughputs alone. Within its limits, our study calculates the quantitative benefit for the recipient from each dollar that CAMTA receives. We encourage medical charities of other types to provide similar information in order to provide the best comparative information to its current and potential donors.

## Figures and Tables

**Table 1:** 15D health state dimensions.

Each dimension is scored on a 5-point ordinal scale to best represent the respondent's present health status:
(i) Mobility (ii) Vision (iii) Hearing (iv) Breathing (v) Sleeping (vi) Eating (vii) Speech (viii) Excretion (ix) Usual activities (x) Mental function (xi) Discomfort and symptoms (xii) Depression (xiii) Distress (xiv) Vitality (xv) Sexual activity

Adapted from http://www.15d-instrument.net.

**Table 2:** Patient demographics for all patients in mission years 2007–2011.

	*µ* (SD) or *n* (%)		
	Enrolled	Lost to follow-up	*p* value
Participants	157	65	

Age	49.1 (10.6)	51.6 (14.8)	0.216
Presurgery PCS	33.9 (6.1)	35.4 (6.6)	0.166
Presurgery MCS	39.9 (8.2)	38.8 (10.2)	0.460

Gender (female)	124 (79.0)	59 (84.3)	0.352
Unilateral	125 (79.6)	65 (93.0)	0.011
Bilateral	26 (16.6)	4 (5.7)	0.025
Staged bilateral	6 (3.8)	1 (0.3)	0.133

**Table 3:** Cost, incremental QALYs gained, and ICER for each surgery type.

Surgery	Mean cost ($CAD)	Mean incremental QALYs (95% CI)		ICER ($CAD/QALY)
		1 year	Lifetime	Lifetime
Unilateral (*n* = 125)	6,042.45 (5893.23–6191.67)	0.081 (0.06–0.11)	1.46 (1.40–1.52)	4,442.77 (4187.76–4697.78)
Bilateral (*n* = 26)	7,229.13 (6876.78–7581.49)	0.12 (0.07–0.16)	2.51 (2.39–2.63)	2,939.49 (2705.00–3173.99)
Staged (*n* = 6)	12,786.08 (11,473.35–14,098.81)	0.14 (0.00–0.29)	2.93 (2.76–3.10)	4,392.23 (3854.22–4930.24)

**Table 4:** Sensitivity analysis for cost of prostheses and of personnel travel and accommodation.

	ICER ($CAD/QALY) for lifetime		
	Unilateral	Bilateral	Staged
Actual	$4,442.77	$2,939.49	$4,392.23
No prostheses cost	$3,411.40	$1,984.32	$1,699.88
Full prostheses cost	$5,515.44	$4,436.35	$5,496.62
No personnel costs	$1,157.53	$1,112.35	$952.90
No personnel or prostheses costs	$402.74	$234.26	$200.68
No personnel with full prostheses costs	$1,755.48	$1,807.97	$1,548.81

## Additional Points

*Implication Statement*. Total hip arthroplasty performed for low-income patients in Ecuador by a Canadian short-stay surgical team resulted in quality of life improvements with favourable cost effectiveness at $2,939 to $4,442 per Quality-Adjusted Life Year (QALY) gained.
